# Sampling and simultaneous determination of volatile per- and polyfluoroalkyl substances in wastewater treatment plant air and water

**DOI:** 10.1007/s00216-016-0072-1

**Published:** 2016-11-25

**Authors:** Ian Ken Dimzon, Joke Westerveld, Christoph Gremmel, Tobias Frömel, Thomas P. Knepper, Pim de Voogt

**Affiliations:** 1 University of Amsterdam, Institute for Biodiversity and Ecosystem Dynamics, Science Park 904, Amsterdam, 1098 XH The Netherlands; 2 Hochschule Fresenius, Institute for Analytical Research, Limburger St. 2, 65510 Idstein, Germany

**Keywords:** Volatile PFAS, GC-MS, WWTP, Influents, Effluents, Air

## Abstract

Volatile per- and polyfluoroalkyl substances (PFASs) are often used as precursors in the synthesis of nonvolatile PFASs. The volatile PFASs, which include the perfluoroalkyl iodides (PFAIs), fluorotelomer iodides (FTIs), fluorotelomer alcohols (FTOHs), fluorotelomer olefins (FTOs), fluorotelomer acrylates (FTACs), and fluorotelomer methacrylates (FTMACs), are often produced starting from the telomerization process. These volatile compounds can be present in the air and water environment and can be transformed into highly persistent perfluoroalkyl carboxylic acids. With the exception of FTOHs, which are well studied, the determination of other volatile PFASs is also of prime importance in studying the sources and fate of PFASs. In this study, a method was developed to determine representative precursor compounds that included PFAIs, FTIs, FTOs, FTACs, and FTMACs in wastewater treatment plant (WWTP) air and water samples. The sampling and sample preparation step involved the use of solid-phase extraction (SPE) cartridges with HLB™ material to enrich the analyte. Gas chromatography with mass spectrometry was employed for the detection and quantification of the analytes. Method validation results showed high linearity and sensitivity in the positive electron ionization-selected ion monitoring mode (+EI-SIM). The absolute instrumental limits of detection were in the range of 0.5 to 2 pg. The method detection limit (MDL) in air was 1 ng/m^3^ with the exception of the FTACs which could be only be detected at concentrations higher than 40 ng/m^3^. The MDL in water was 10 ng/L. Direct spiking of the cartridges and analyte introduction by volatilization from the glass surface onto the SPE material had recoveries between 86 and 100%. The volatile PFASs were shown to readily partition into the air rather than into water. Consequently, large losses in the amount of PFASs were observed when these were spiked into the water.

**Graphical abstract:**
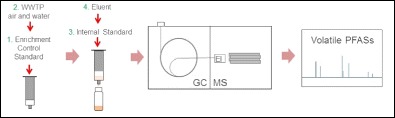
Wastewater treatment plant air and water samples were passed through HLB™ solid-phase materials. The eluates were injected onto a GC-MS system to simultaneously determine the volatile PFASs.

Wastewater treatment plant air and water samples were passed through HLB™ solid-phase materials. The eluates were injected onto a GC-MS system to simultaneously determine the volatile PFASs.

## Introduction

The interest in determining the different poly- and perfluorinated alkyl substances (PFASs) in the environment has increased rapidly in recent years. These compounds have been labeled as pollutants due to their various harmful effects that spring from their unique properties, such as their low degradability. Perfluoroalkyl carboxylic acids (PFCAs) and perfluoroalkane sulfonic acids (PFSAs), particularly their C_8_ homologues perfluorooctanoic acid (PFOA) and perfluorooctane sulfonic acid (PFOS), have been the subject of many studies—their occurrence, fate, and distribution in different samples and environmental compartments [1]; their biodegradability and bioaccumulation [2, 3]; and their toxicities to different organisms [4, 5]. In controlling these substances, there is a need to identify their possible sources. One potential source is the physico-chemical and biological transformation of other PFASs [6–8].

**Fig. 1:**
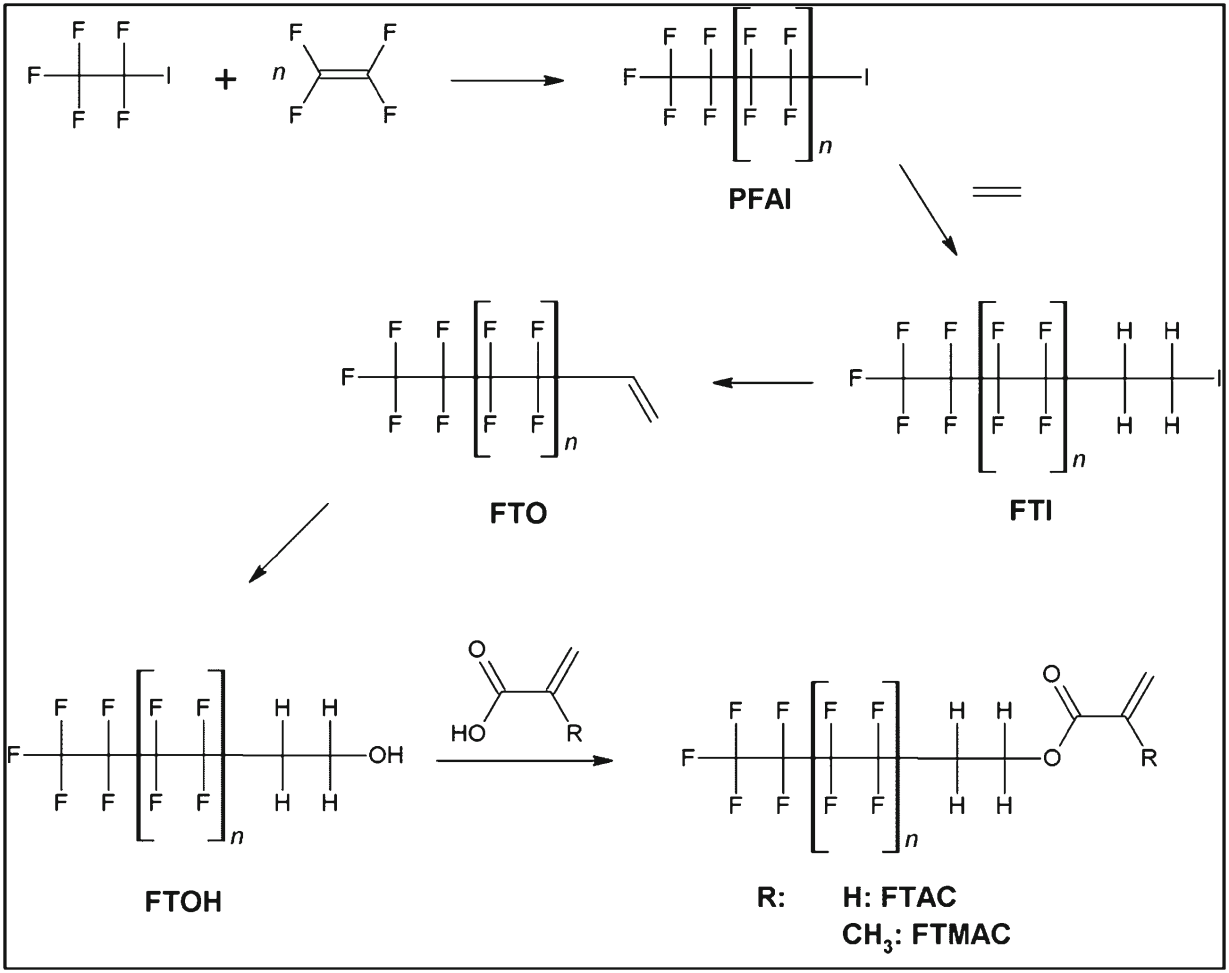
Synthesis of volatile PFASs starting from the telomerization process (*n* = 2 to 6)

The majority of PFASs are synthesized starting from the telomerization process (Fig. 1). It involves first, a UV-catalyzed radical reaction of trifluoromethyl iodide producing perfluoroalkyl iodides (PFAI) of varying number of carbon atoms [1, 9]. Then, the PFAIs are further reacted forming fluorotelomer iodides (FTIs), fluorotelomer alcohols (FTOHs), and fluorotelomer olefins (FTOs). Monomers like acrylates and methacrylates are esterified with FTOHs producing fluorotelomer acrylates and methacrylates (FTACs and FTMACs).

The unreacted species in the synthesis of PFASs can be released into the environment during the manufacturing process as part of the industrial waste. A survey of the area around a fluorochemical manufacturing plant in China revealed high concentrations of even numbered carbon PFAIs and FTIs in the air. The study also showed that these precursor compounds are easily volatilized and only the longer chains are detected in the soil samples [10]. The unreacted species are also present in the final products as residuals. For example, in a study by Dinglasan-Panlilio and Mabury, all the fluorinated materials tested contained unbound fluorinated alcohols at a level between 0.4 and 3.8% of the dry mass of the samples. The authors suggested that the residuals can be potential sources of FTOHs released into the environment [11].

Given the pivotal role of the volatile PFASs in the synthesis of the majority of the other PFASs, the detection and quantification of these compounds would be important in accounting the sources of PFASs in the environment [6, 12, 13]. In spite of this, volatile PFASs except the FTOHs are not usually measured in environmental samples as indicated by the limited availability of literature sources [10, 14–16]. PFAIs, FTIs, FTOs, FTACs, and FTMACs, given their high F/C ratios, are not ionizable using some common atmospheric pressure ionization techniques in mass spectrometry (MS). Another challenge in the measurement of the abovementioned compounds is their high volatility and extremely low water solubility, making it difficult to prepare aqueous standard solutions. Most of the widely studied PFASs like the PFCAs, PFSAs, and FTOHs are usually determined using liquid chromatography (LC) with electrospray ionization (ESI) or atmospheric pressure chemical ionization (APCI)-MS detection. Thus, if volatile PFASs are also to be determined, then a separate method needs to be developed. Other problems that can arise in measuring volatile PFASs include their handling. FTACs and FTMACs can stick to the glass containers while PFAIs are unstable.

In this paper, we report on the development and validation of a complete method to determine the volatile PFASs except FTOHs in industrial and municipal wastewater treatment plant (WWTP) air and water. A sampling and enrichment method was developed for the volatile PFASs in the air samples using HLB™ solid-phase extraction (SPE) cartridges. The collected influents and effluents were also enriched using HLB™ SPE cartridges. Analyte separation was done using gas chromatography (GC) while MS with electron ionization (EI) ion source was utilized to detect and quantify the analytes. FTOHs were determined in a separate, parallel study using LC-tandem MS (Gremmel et al. 2016 *Development of an LC-MS/MS multi-method for the determination of perfluoroalkyl and polyfluoroalkyl substances in environmental samples. Anal Bioanal Chem. in review*). We also report on the difficulty we encountered in assessing the accuracy (more specifically, trueness) of the method for WWTP influents and effluents. A pseudo-partitioning experiment, made possible using the developed method, was done to verify the initial inferences. The work presented in this paper is part of a project that investigated the presence of 65 PFASs in different WWTPs and that resulted in an extended report that can be found on the German Environmental Agency’s website [17].

## Materials and methods

### Materials, solvents, and standards

The SPE set-up consisted of a 24-port Visiprep™ vacuum manifold (Supelco, Pennsylvania, USA) connected to ME IC vacuum pump (Vacuubrand GmnH, Wertheim, Germany), and Oasis® HLB™ SPE cartridges with 60 mg sorbent material and 3 cm^3^ volume (Waters Corporation, Milford, USA).

The following analytes were included in the method: 1H,1H,2H-perfluoro-1-octene (6:2-FTO); 1H,1H,2H-perfluoro-1-decene (8:2-FTO); 1H,1H,2H-perfluoro-1-dodecene (10:2-FTO); perfluoro-n-hexyl iodide (PFHxI); perfluorooctyl iodide (PFOI); perfluorodecyl iodide (PFDI); 1H,1H,2H,2H-perfluorohexyl iodide (4:2-FTI); 1H,1H,2H,2H-perfluorooctyl iodide (6:2-FTI); 1H,1H,2H,2H-perfluorodecyliodide (8:2-FTI); 1H,1H,2H,2H-perfluorooctyl acrylate (6:2-FTAC); 1H,1H,2H,2H-perfluorodecyl acrylate (8:2-FTAC); 1H,1H,2H,2H-perfluorooctyl methacrylate (6:2-FTMAC); and 1H,1H,2H,2H-perfluorodecyl methacrylate (8:2-FTMAC). The following compounds were used as control standards: 1H,1H,7H-dodecafluoroheptyl iodide (7H-6:1-FTI) and 1H,1H-perfluorooctyl acrylate (7:1-FTAC). 1H,1H,2H,2H-perfluoro-7-methyloctyl iodide (7Me-6:2-FTI) was used as GC injection internal standard (IS). The analytes and control standards were purchased as pure reagents (95 to 99% purity) from ABCR GmbH (Karlsruhe, Germany). Methanol (ULC/MS grade, 99%) and *n*-pentane (LC/MS grade, 99%) purchased from Biosolve (Valkenswaard, The Netherlands) were used as solvents in the preparation of working solutions and calibration solutions. Two sets of calibration solutions were prepared. Calibration set A has a concentration range from 5 to 60 ng/mL while calibration set B has a range from 50 to 800 ng/mL.

### Sampling sites and set-up

The air and water samples were collected from different industrial and municipal WWTP in the Netherlands and in Germany. The air sampling set-ups were directly placed above the compartments where the influent enters the WWTP. The sampling of influent, air and effluent was scheduled in such a way that there was correspondence in the collected samples (i.e., lag times were taken into account).

### Air sampling and enrichment

The HLB™ cartridges were conditioned twice with 2 mL MeOH and dried under a stream of N_2_ gas for 15 min. The cartridges were spiked with 20 μL enrichment control standard solution (1.0 μg/mL 7H-6:1-FTI and 7:1-FTAC in methanol) and were dried again under a stream of N_2_ for 5 min at a flow rate of 100 mL/min. Air sampling was carried out using a low-volume air sampling device (type GS 312, DESAGA, Germany, Heidelberg). The cartridge was attached to a membrane pump with a low-volume flow controller and a silica moisture trap. The sampling was undertaken at an air flow rate of 1 to 2 L/min for 24 h. The final volume of the air that was passed through the cartridge was recorded. The set-up was placed over the coarse waste filtration area where the influent enters the WWTP.

**Fig. 2:**
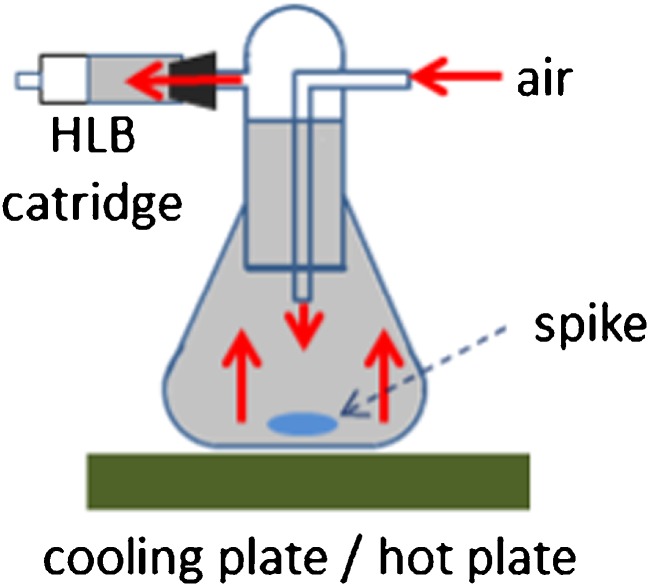
Set-up for the spiking of the analytes by volatilization

To study the enrichment of volatile PFASs in the air into the HLB™ cartridge, 20 μL of 1.0 μg/L volatile PFASs stock solution was spiked into a 300-mL Erlenmeyer flask with connection joints that was previously placed on ice (Fig. 2). The flask was then sealed with a stopper with two openings connected to a SPE cartridge with enrichment control standard and to an air source. The flask was then transferred to a sand bath that was pre-heated to 60 °C. Air was allowed to flow into the flask at ∼100 mL/min flow rate for 20–30 min. The cartridges were stored at 20 °C until GC-MS analysis.

### Water sampling and enrichment

The HLB™ cartridges were conditioned twice with 2 mL methanol, and twice with 2 mL sub-boiled water. The water samples were filtered using a suction filtration set-up with Whatman GF/F glass microfiber filter (0.7 μm pore size, 4.7 cm diameter). Aliquots of 100 mL water samples were transferred into separate brown bottles and were spiked with 20 μL enrichment control standard solution. The water samples were passed through immediately after spiking into the conditioned SPE cartridges using the SPE-manifold that was connected to a vacuum. The flow of water was maintained at 0.05 to 0.1 mL/s (1–2 drops/s). The cartridges were then dried using a stream of N_2_ gas for 15 to 30 min. The enriched SPE cartridges were wrapped with aluminum foil and were stored at −20 °C until GC-MS analysis.

### GC-MS analysis

The cartridges were first brought to room temperature. A 20 μL of the GC injection IS solution (1 μg/mL of 7-Me-6:2-FTI in *n*-pentane) was spiked directly into the cartridges. The cartridge was then immediately loaded with 2 mL *n*-pentane to elute the analytes and the control and internal standards. Only the first 1 mL of eluate was collected in a 2-mL vial with 0.5 mL graduation and the remainder was discarded.

One microliter of the sample or standard solution was introduced into the GC-MS (Thermo Fisher; Trace GC 2000/Trace MS; Waltham, USA) via an autosampler (PAL Combi-xt; CTC Analytics; Zwingen, Switzerland). The injector and the oven parameters were initially developed. After optimization, the following GC parameters were adapted in the final method: Injector port: 180 °C, splitless mode with surge pressure of 200 kPa for 0.50 min; Carrier gas: Helium with 1.8 mL/min flow; column: Restek VMS (30 m length, 0.25 mm i.d., 3.0 μm film thickness, Bellefonte, USA); oven temperature: set at 35 °C initially for 2 min, increased to 45 °C at a rate of 2 °C/min; increased to 100 °C at a rate of 10 °C/min; increased again to 110 °C at 1 °C/min; finally increased to 240 °C at 30 °C/min and was held at final temperature for 1 min [17].

The EI source voltage was set at 70 eV, and the mass analysis was done in a scheduled selected ion monitoring (SIM) mode with a constant scan time of 0.40 s. The quantifier and qualifier ions were chosen for each analyte based on the criteria of high ion intensity and uniqueness [17]. Standard perfluorotributylamine was used to tune the MS. Data processing was done using the Qual browser of the Xcalibur software ver. 2.2 (Thermo Fisher). During method development, peak identification was carried out by comparison with the mass spectra and retention times of single standards and with the NIST mass spectral library (ver. 2).

### Pseudo-partitioning experiment

**Fig. 3:**
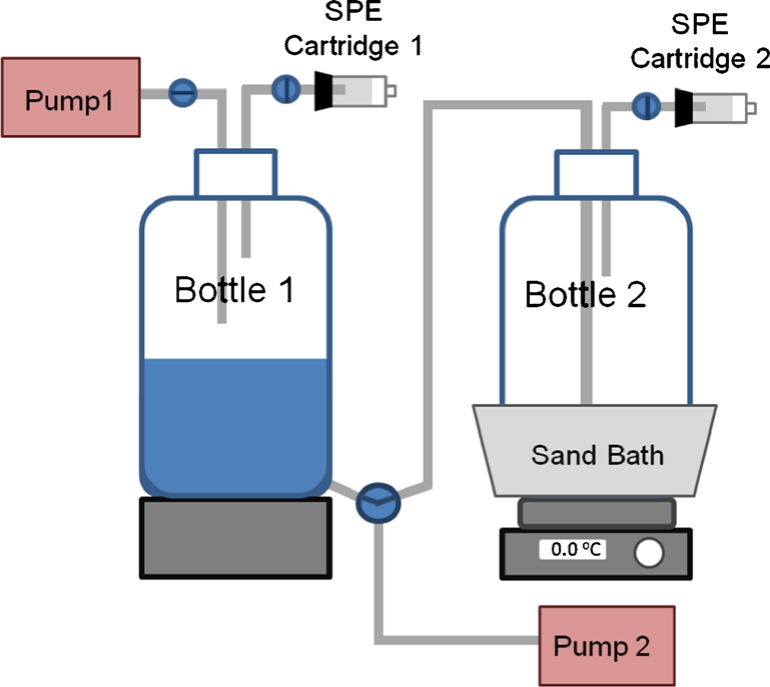
Set-up of the pseudo-partitioning experiment

The pseudo-partitioning experiment set-up consisted of two 4-L bottles (bottle 1 and bottle 2). The two bottles were connected to two pumps and two HLB™ SPE cartridges as shown in Fig. 3. Initially, bottle 1 was filled with 2 L of water. A known volume of the methanolic volatile PFASs solution was added into the water while the tip of the pipet was submerged intentionally into the water. The spike contained 20 ng of each volatile PFAS. Bottle 1 was then closed and was equilibrated for 24 h. After equilibration, the water was pushed toward bottle 2 by introducing air in bottle 1 via aeration pump 1. The connection was promptly closed to prevent transfer of gas from bottle 1 to bottle 2. The gases including the volatile PFASs that partitioned into the air in bottle 1 were forced through SPE cartridge 1 using air pumped in by aeration pump 1. At the same time, bottle 2 was heated up to 60 °C in a sand bath while air was bubbled through the water using aeration pump 2. The air and the volatile compounds including the PFASs that were bubbled out were forced through cartridge 2. SPE cartridges 1 and 2 were processed for GC-MS as described in “GC-MS analysis” section.

## Results and discussion

### Development and validation of the GC-MS method

A GC-MS method was developed to identify and quantify the volatile PFASs except FTOHs. Aside from being well studied, FTOHs have different MS ionization characteristics. GC is a fitting method to separate and determine highly volatile compounds. Additionally, FTOs, FTIs, PFAIs, FTAC, and FTMAC were found to be easily ionizable by EI and chemical ionization (CI). However, unlike the FTOHs, the volatile PFASs in this study are not ionizable by ESI and APCI limiting the application of liquid chromatography.

The GC method was optimized in terms of the column oven temperature and the injection system. The final oven program consisted of multiple steps with different rates of temperature ramping that allowed better separation of the different PFASs. Splitless injection (at 180 °C) was chosen over on-column injection (at 35 °C) because of the tiny but uncontrollable system leak in the latter. The leak decreased the injection repeatability and method sensitivity for 6:2-FTO and PFHxI, which were the most volatile from among the analytes. In splitless injection, however, the peaks from the early eluting analytes were broader resulting to a slight decrease in sensitivity. Overall, splitless injection had better performance characteristics.

Three ionization techniques were tested and explored: EI, positive chemical ionization (PCI), and electron-capture negative ionization (ECNI). All the PFASs in the study were ionized using EI; however, the ion counts of the molecular ions (radical cation) were very low. The fragment ions produced by EI were results of the cleavage of C-C bonds; the ω-C-C bond was the easiest to be broken producing CF_3_^+^ fragment with *m*/*z* of 69. This fragment was detected in all the PFASs that were analyzed albeit in varying relative ion count. The FTOs were heavily fragmented to low molecular weight common ions with *m*/*z* of 77 and 131. This indicated that the electron energy of 70 eV caused heavy fragmentation of the analytes. The electron energy of 45 eV was tested in an attempt to reduce the degree of fragmentation and to obtain ions with higher molecular weight. This, however, did not result to a significantly different mass spectrum compared to that from 70 eV. Another way to reduce fragmentation was to employ chemical ionization as an alternative method. PCI and ECNI, with methane as the reagent gas, were thus performed. The results from these chemical ionization techniques were compared to that of EI. In PCI, the prominent ions were the protonated molecule ([M + H]^+^), and the C_2_H_5_^+^ adduct ([M + C_2_H_5_]^+^). The elimination of HI and HF from the protonated molecule produced the [M-I]^+^ and [M-F]^+^ fragment ions, respectively. ECNI, like EI, was characterized by heavy fragmentation. The signal from *m*/*z* 127 created a very high baseline in the total ion chromatogram (TIC) of ECNI. The counts of ions in PCI and in ECNI (except for the ion counts of I^−^ from PFAIs and FTIs) were lower compared to those in EI by three to four orders of magnitude.

The peaks in the chromatogram were identified using various means that included (1) comparison of the EI-mass spectrum of the unknown peak and the EI-mass spectrum of the compound that appeared when the mass spectral database (using the NIST library) similarity search function was performed; (2) comparison of retention times and EI-mass spectra of the standard mix and the single standard solutions; (3) comparison of the mass spectra from EI, PCI, and ECNI; and (4) evaluation of the fragmentation patterns in the EI-, PCI-, and ECNI-mass spectra versus the chemical structure of each compound.

**Fig. 4:**
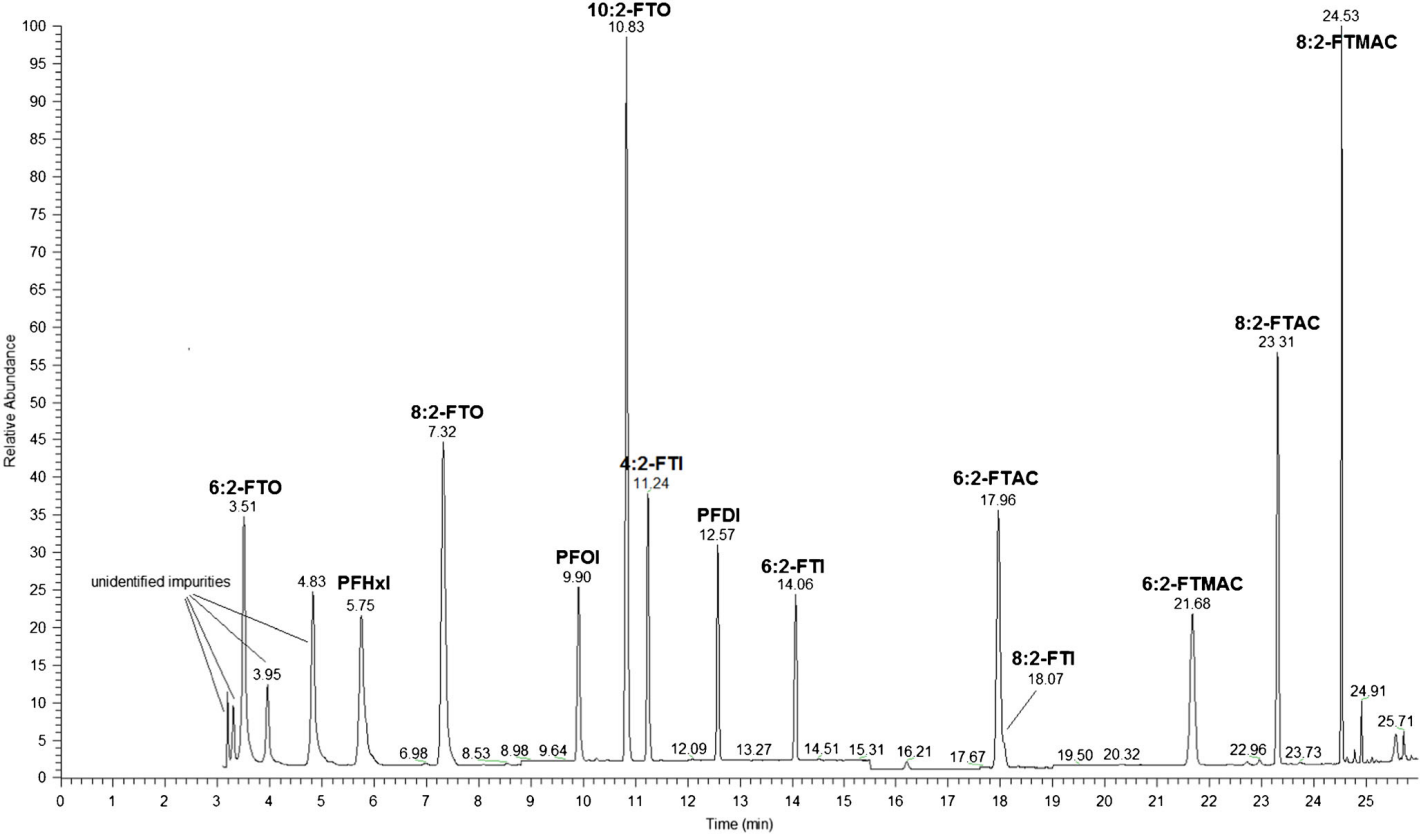
Total ion chromatogram of 600 ng/mL standard solution determined by +EIMS (70 eV electron energy) in SIM mode

SIM was employed for the quantitative determination of the volatile PFASs. Two or three ions with the most intense signals were chosen for the quantification and qualification of the analytes. Figure 4 shows the TIC of a 600 ng/mL standard solution generated using the optimized oven program with the +EI detection in the selected ion monitoring mode. The compounds 6:2-FTAC and 8:2-FTI were not fully separated as shown in the TIC. However, the quantification were done using ions that are not common to both.

The validation results of the developed GC-EIMS method is shown in Table 1. As a criterion to verify the identity of a compound, the repeatability of the area ratio of the qualifier ion to the quantifier ion per compound at very low concentration (1–20 ng/mL) was tested. The results show that except for 6:2-FTO, the area ratios of the qualifier ion to the quantifier ion have RSD of less than 20% across a concentration range from 5 to 60 ng/mL. The qualification limit in ng/mL is the lowest concentration in which the ratio of the qualifier ion to the quantifier ion can be calculated. At a concentration below this limit, either the qualifier ion or the quantifier ion, or both are non-detectable.

**Table 1:** Summary of the GC-EIMS method performance characteristics

Analyte	Selectivity area ratio criterion			Average sensitivity (*n* = 5)		Average coefficient of determination (*R* ^2^, *n* = 5)		Repeatability of injection given as % RSD (*n* = 10)		Absolute LOD (pg)
	Area ratio quantifier ion to the qualifier ion	RSD (%)	Absolute qualification limit (pg)	Average	RSD (%)	Absolute area	Normalized against IS^a^	(Absolute area)	(Normalized against IS^a^)	
6:2-FTO	2.90E-02	27	5	3.2E + 04	6	0.9962	0.9968	6.6	4.7	0.3
8:2-FTO	3.80E-02	5	5	5.0E + 04	6	0.9966	0.9989	6.7	3.7	0.3
10:2-FTO	4.60E-02	7	2	5.6E + 04	5	0.9951	0.9995	7.9	2.2	0.3
PFHxI	2.60E + 00	10	2	4.9E + 03	7	0.9950	0.9985	7.9	4.1	0.6
PFOI	1.80E-01	18	1	1.4E + 03	6	0.9922	0.9981	9.6	5.8	1
PFDI	5.40E-02	8	2	5.8E + 02^b^	9^b^	0.9939^b^	0.9975^b^	11	5.6	1
4:2-FTI	4.60E-01	5	1	6.9E + 03	6	0.9955	0.9996	7.3	1.9	0.3
6:2-FTI	3.20E-01	4	1	5.0E + 03	6	0.9947	0.9996	8.6	1.2	0.3
8:2-FTI	3.90E-01	13	2	2.1E + 03	6	0.9929	0.9991	8.7	3.0	1
6:2-FTAC	1.00E-01	12	2	3.1E + 04	5	0.9972	0.9971	8.2	5.4	1
8:2-FTAC	1.00E-01	8	2	2.6E + 04	4	0.9946	0.9992	6.0	4.6	0.6
6:2-FTMAC	7.40E + 00	10	1	1.9E + 03	4	0.9907	0.9975	11.8	6.2	1
8:2-FTMAC	1.00E + 01	17	1	1.4E + 03	4	0.9956	0.9995	7.8	3.3	0.6

^a^7Me-6:2-FTI was used as an internal standard

^b^The 2 ng/mL standard was omitted in the evaluation of the calibration curve of PFDI

Five sets of mixed calibration standards were prepared and analyzed. Each calibration set has six solutions with analyte concentrations ranging from 2 to 60 ng/mL. In addition, the calibration solutions were spiked with the GC injection IS to a final concentration of 20 ng/mL. The calibration curve for PFDI was an exception and starts from 5 ng/mL instead of 2 ng/mL. The sensitivity of the method to all analytes was good as indicated by high slope values. The coefficients of determination for both calibration solution series with and without internal standard were greater than 0.99. The area ratio of the analyte to the IS was more repeatable than when only the area of the analyte was used and therefore, in all quantitative work, the internal standard was used. The absolute instrumental LODs were between 0.3 and 1 pg.

### SPE enrichment of air

**Fig. 5:**
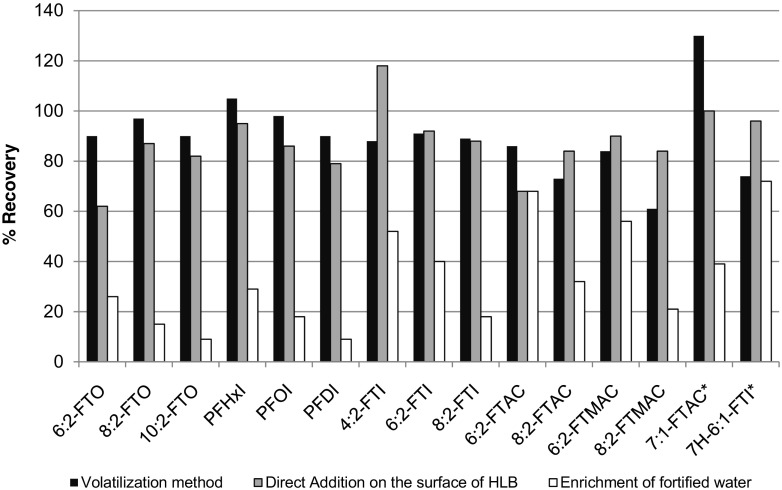
Percent recoveries of the volatile PFASs (20 ng each) by HLB enrichment using three different techniques; *n* = 4 for volatilization method, and *n* = 1 for direct addition and enrichment of fortified water (*7:1-FTAC and 7H-6:1-FTI are enrichment control standards)

The performance of HLB™ SPE cartridges to enrich the analytes in the vapor phase and those dissolved in water was studied. Three techniques were investigated: (1) volatilization of the analyte using the set-up shown in Fig. 2 and described in “Air sampling and enrichment” section; (2) direct addition of the methanolic solution on the top surface of the HLB™ material in the SPE cartridge; and (3) enrichment of fortified water. The amounts of volatile PFAS used for the test were 200 and 20 ng. The percent recoveries of the three techniques for the 20 ng volatile PFAS are summarized in Fig. 5.

The volatilization and the direct addition methods have recoveries between 60 and 120% for all the analytes. Volatilization method was better compared to the direct addition method for FTOs, PFAIs, and FTIs. The percent recoveries of the mentioned compounds are between 80 and 100%. The percent recoveries of FTACs and FTMACs were initially inconsistent and problematic, probably due to desorption on the glass surface. The percent recoveries of the FTACs and FTMACs were improved by heating the bottom of the flask to 60 °C while streaming air.

Direct addition of the methanolic standard solution on the surface of SPE material yielded lower percent recoveries for FTOs, PFAIs, and FTIs (except 4:2-FTZI). The difference in the percent recoveries using the two methods is nearly 30% for 6:2-FTO, the most volatile homologue and the earliest eluting peak. The losses could be due to the desorption of the volatile compounds from the SPE material by the methanol vapor at a short instance. A 20 μL portion of the standard solution in methanol was spiked.

The compounds 7:1-FTAC and 7H-6:1-FTI were added as enrichment control standards. We define enrichment control standards as compounds that are deliberately spiked/added into the sample (in case of water) or onto the SPE material (in case of the air samples) to monitor the efficiency of the enrichment process. Figure 5 also shows that the percent recoveries for 7:1-FTAC and 7H-6:1-FTI were 130 and 70%, respectively, using volatilization method, and 100 and 96%, respectively, by direct addition.

The third method used to study the efficiency of HLB was by enrichment of fortified water samples. The result of this method is discussed in “SPE enrichment from influent and effluent” section.

The method developed for air was applied to the analysis of the air above the influent in an industrial and a municipal WWTP. Approximately 1000 to 3000 L of air was passed through HLB™ SPE cartridges at different days. The actual volumes of air were calculated and used in the determination of analyte concentration. None of the analytes was detected in the air above the municipal WWTP influent. The method detection limit was approximately 1 ng/m^3^ air. Due to the high background signal at *m*/*z* 69, the method detection limit of FTACs was higher at 40 ng/m^3^ air. In a parallel study, the presence of FTOHs in air above the same WWTP was confirmed at levels of 0.1–15 ng/m^3^ [17]. The non-detection of the volatile PFASs in the municipal waste is expected because these compounds are not the final products themselves. They could be present in domestic products as synthetic residues. Their entry into the water system and their fate in the municipal WWTP are less likely due to their extremely low solubility in water and high volatility.

Substantially high amounts of 6:2-FTMAC per liter of air were detected in the air above the industrial WWTP influent as shown in Table 2. FTOs were also detected at this location but in lesser concentration, whereas none of the other volatile compounds were detected. Unlike in municipal WWTP, detection of volatile PFASs in industrial WWTP is very likely especially if there is a nearby PFAS manufacturing plant. Upon entry into the WWTP, most of the volatile PFASs influent would be lost into the air. More losses are expected if the influent is subjected to aeration and heating. The compounds detected reflect the nature of the industrial activity in the vicinity. The detection of high amounts of 6:2-FTMAC could be an indication in the shift to C6 chemistry in the PFAS production.

**Table 2:** Volatile PFASs detected in the air above the industrial WWTP influent

Detected analyte	Volatile PFASs concentration in each sampling day (ng/m^3^ air)								Method detection limit (ng/m^3^)	Method quantification limit (ng/m^3^)
	1	2	3	4	5	6	7	8		
6:2-FTO	n.d.	n.d.	115	9.5	6.7	14.6	6.1	2.7	1	3
8:2-FTO	n.d.	n.d.	7.4	2.9	<LOQ	6.6	6.5	<LOQ	1	3
10:2-FTO	n.d.	215	31.6	6.4	12.3	56.7	37.8	10.7	1	3
6:2-FTAC	<LOQ	n.d.	n.d.	n.d.	<LOQ	<LOQ	<LOQ	<LOQ	40	120
8:2-FTAC	n.d.	n.d.	n.d.	n.d.	645	1603	329	163	40	120
6:2-FTMAC	1370	33,100	2340	369	471	1870	1200	854	1	3
8:2-FTMAC	n.d.	22.3	n.d.	n.d.	n.d.	7.2	n.d.	n.d.	1	3

*n.d.* not detected

### SPE enrichment from influent and effluent

As was mentioned in “SPE enrichment of air” section, the third method used to study the efficiency of HLB was by enrichment of fortified water samples. A 20 ng amount of each of the analytes was introduced into 200-mL deionized water. The fortified water was then passed through the HLB cartridge. The percent recoveries of the analytes and control standards were variable and not greater than 75%. The FTOs, PFAIs, 8:1-FTI, and 8:1-FTMAC have recoveries less than 30%. Aliquots of municipal WWTP influent and effluent in which the volatile PFASs were not detected were also tested. The recoveries were similarly low. There are two possible explanations: (1) the HLB™ material was not efficient enough to trap the dissolved volatile PFASs in water; or (2) the volatile PFASs were lost nearly instantaneously into the air after spiking. As will be discussed in “Water-air partitioning of the volatile PFASs” section on the results of the water-air partitioning experiment, volatile PFASs easily partition into the air above the water. This is due to the highly hydrophobic and very volatile nature of these compounds. This makes calculation of percent recovery and evaluation of analytical trueness impossible. Also, the control standard added to the water before enrichment cannot correct for any error related to the sample preparation and the measurement because the magnitudes of losses due to partitioning and matrix effect for each compound are not proportional.

**Fig. 6:**
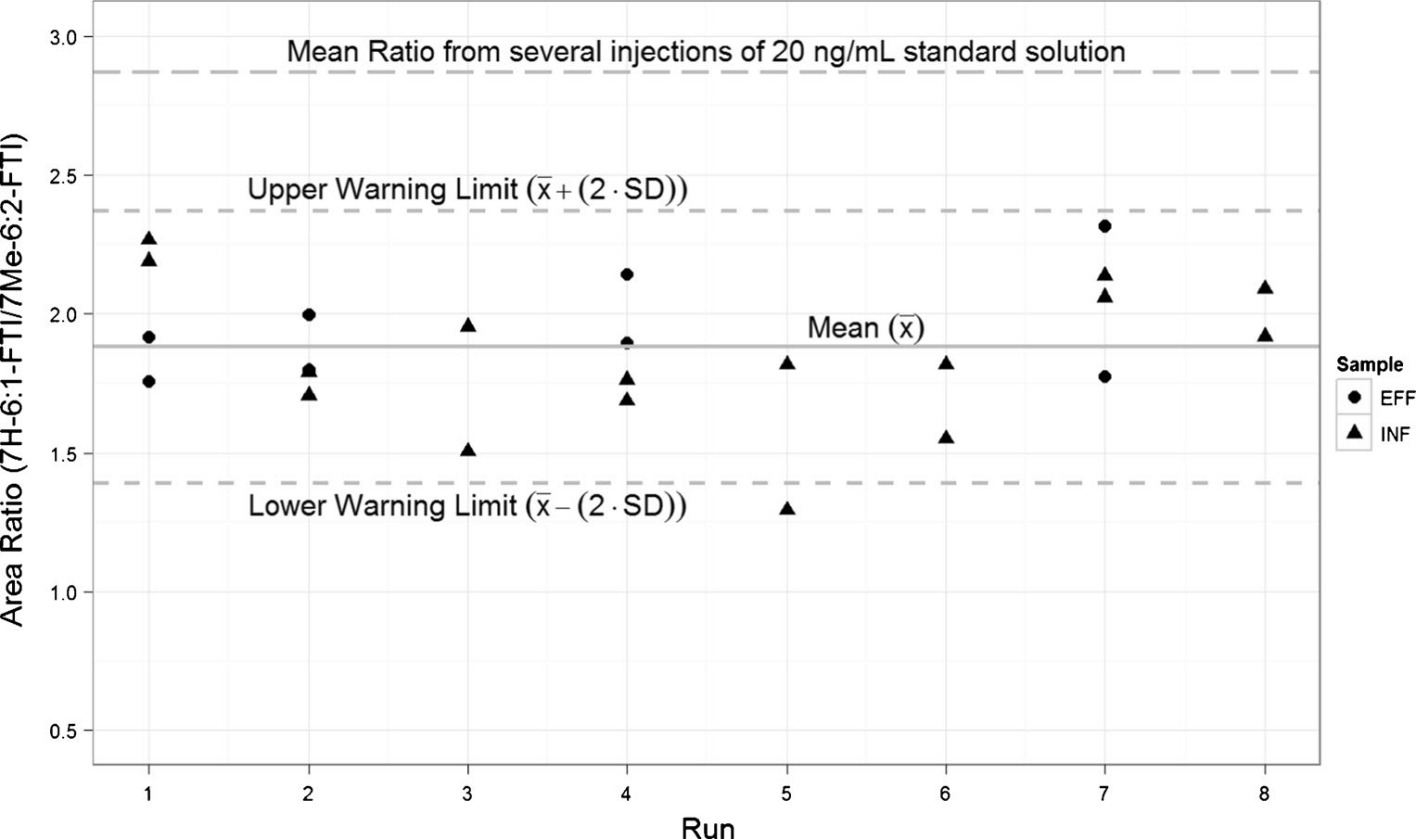
Control chart of the area ratios of enrichment control standard (7H-6:1-FTI) to the GC injection IS (7Me-6:2-FTI) recovered from spiked (20 ng) effluent (*EFF*) and influent (*INF*)

One way to assess and control the quality of measurements of the volatile PFASs in water samples is to add enrichment control standards into the water samples. The area ratio of the enrichment control standard to the GC injection control standard can then be calculated from the chromatograms. The area ratios of the control standards can be plotted in a control chart with estimated warning and critical limits. Figure 6 shows the control chart generated for the municipal WWTP influents and effluents spiked with the control standards. Even with losses due to partitioning, when the enrichment process is strictly controlled, the precision of the method can be improved. The results for influents and effluents can be taken as acceptable if the ratio of the control standards falls within the control limits.

In implementing the method, the influent and effluent samples were always spiked with the control standards just prior to enrichment. This limits the losses due to fast partitioning into the headspace. It is assumed that the amount of the enrichment control standards that remained will be within the control limits in any of the water samples when the procedure is repeated uniformly to all samples. When the amount of the control standards relative to the GC injection IS was outside the control limits (indicated by the area ratios), the result was reviewed or the analysis repeated. It can be noted that this step is a qualitative assessment and has no implication in the calculation of the concentration of volatiles present in the water samples.

The method developed for water samples was used in the analysis of influents and effluents from a municipal and an industrial WWTP. The compounds were not detected in the municipal wastewater samples. The MDL was 10 ng/L. In the parallel study, the FTOHs were also not detected in the municipal wastewater samples. The MDL of FTOHs was varying between 5 and 15 ng/L [17].

Only 6:2-FTMAC was found to be present in high amount (200 to 4600 ng/L) in the industrial WWTP influents. This is consistent with the results of the analysis of industrial WWTP air. No analyte was detected in the wastewater effluents.

### Water-air partitioning of the volatile PFASs

The pseudo-partitioning experiment was developed to confirm the initial observation that there were high losses in the recovery of the volatile PFASs that were spiked in water. This experiment including the set-up described in Fig. 3 was not designed for the accurate determination of partition coefficients and Henry’s law constants. The experiment is limited in the following ways: (1) the actual time when equilibration will be reached was not studied and the 24-h period equilibration time was not optimized; (2) the partitioning between air and glass surface, and between water and glass surface were not taken into account; and (3) there is a possibility of disturbance in the equilibrium during the transfer of the water from bottle 1 to bottle 2. Despite these limitations, the obtained ratios can be used to gain insights into the different processes involved.

**Fig. 7:**
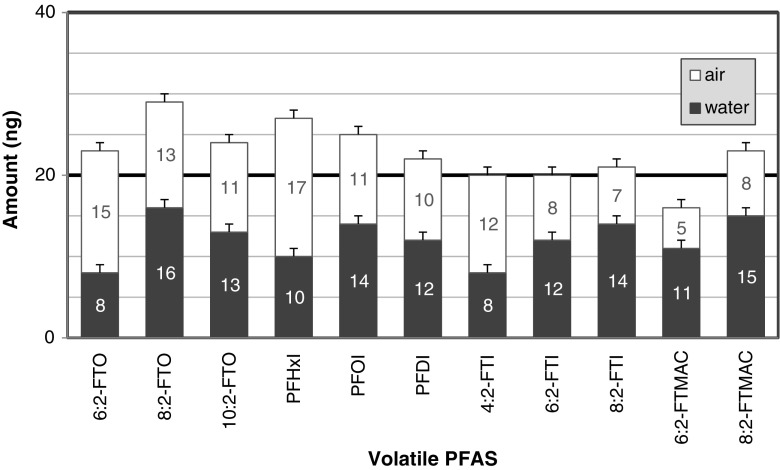
Water-air partitioning of the 20 ng volatile PFASs spiked into 2 L of water with a headspace of 2 L of air after 24 h of equilibration at 20 °C. The positive *error bar* is 1 SD (*n* = 2)

The results of the pseudo-partitioning experiment are presented in Fig. 7. Between 30 and 60% of each of the volatile PFAS added into the 2 L water was transferred into the 2 L air. The total amounts of volatile PFAS in air and in water were greater than the initially added amount of 20 ng with the exception of the FTIs and 6:2-FTMAC. There could have been some cross-contamination and carry over from the previous runs during the method and set-up development. The FTACs were excluded in the figure because of very high carry-over. The FTACs from preceding runs were adsorbed on the surface of the glass bottles and then are desorbed in high amounts in one of the proceeding runs. The outcome is a large amount of the FTAC in the air and water in the runs. The ratios observed for each compound are consistent with the results of the HLB recovery studies of fortified water samples shown in Fig. 5. This experiment confirms the high theoretical Henry’s Law values calculated for the volatile PFASs which can be attributed more to their high hydrophobicity.

## Conclusion

Studying the occurrence of volatile PFASs in environmental samples such as WWTP air and water is important to account for the different sources of the persistent and toxic nonvolatile PFASs like PFCAs and PFSAs. A GC-EIMS method was developed to separate, detect, and quantify the abovementioned compounds simultaneously. The instrumental method was sensitive and has linear response (*R* ^2^ > 0.99) at concentrations from 5 to 800 ng/mL. The absolute instrumental LOD was in the range between 0.3 to 1 pg. Additionally, SPE using HLB cartridge was developed to enrich the analytes from the air and water samples.

It was shown that due to their highly volatile and highly hydrophobic nature, the volatile PFAS partitioned more into the headspace than in water. This creates a problem when the analytes are to be spiked in water to study the efficiency of SPE method for water and hampers the evaluation of the method accuracy. Upon spiking, the analytes were lost almost instantaneously from the water sample to the headspace. As a consequence, their corresponding percent recoveries were reduced by a third to more than a half. The low recoveries cannot therefore be associated with the poor efficiency of the SPE cartridges in enriching the analytes.

Given the problem of analyte loss due to partitioning, the following quality control procedure was done and is recommended in place of percent recovery calculations to control the quality of measurements: Prior to enrichment, enrichment control standards should be spiked into the water samples. A control chart with warning limits based on method validation studies and the preceding valid runs can be set-up based on the areas of the enrichment and injection control standards. The measurement can be judged reliable if the area ratio falls between the upper and lower warning limits.

The developed and validated method reported in this paper was used in studying the fate of PFASs in WWTP that will be published separately.
